# Supplementary material for: Structure-Affinity Correlations and Separable Optical Activity in Carbon Nanotube Protein Coronas
Source: ACS Nano. 2026 Oct 29;20(44):30295–306. doi: 10.1021/acsnano.6c15740 (PMC13758394; doi:10.1021/acsnano.6c15740)
Supplement: Supplementary file 1 [file nn6c15740_si_001.pdf]

Supporting Information for

Structure-affinity correlations and separable optical activity in carbon nanotube protein coronas

Jacob Miller, Alexis Coscia, Aceer Nadeem, Mijin Kim*

School of Chemistry and Biochemistry, Georgia Institute of Technology, Atlanta, GA
*Corresponding author: mkim445@gatech.edu

Table of Contents

Supplementary Methods……………………………………………………………………..……….2
Supplementary Note 1……………………………………………………………..………….3
Supplementary Note 2……………………………………………………………..………….5

Figure S1 6
Figure S2 7
Figure S3 8
Figure S4 9
Figure S5 10
Figure S6 11
Figure S7 11
Figure S8 12
Figure S9 12
Figure S10 13
Figure S11 15
Figure S12 14
Figure S13 16
Figure S14 17
Figure S15 18
Figure S16 19
Figure S17 20
Figure S18 21
Figure S19 22

Table S1 23
Table S2 24
Table S3 25
Table S4 25
Table S5 26
Table S6 27

Supplementary References……………………………………………………………….27

Supplementary Methods: Reanalysis of Goerzen et al. fluorescence titration data

We reanalyzed the fluorescence titration data reported by Goerzen et al.[1] for DSPE-PEG wrapped 3,4,5-trifluoroaryl QWNTs (Figures S17-S19). For the enrichment comparison, the 22 proteins present in both the Goerzen dataset and our corona proteomics were grouped as enriched (n = 11) or depleted (n = 11) by their corona enrichment. The dataset comprised triplicate measurements for plasma proteins across seven concentrations (0.015–246 µg/mL) and four spectral features per protein: the E_{11} and E_{11}^{-} wavelength shifts (*dwl*, *dwl** in nm) and the E_{11} and E_{11}^{-} intensity changes (*dint*, *dint** in %). Individual replicate values were used throughout the analysis.

Molecular weights for the protein standards used in the titrations were taken from the source and vendor information reported in the original study, and each protein was converted from mass concentration to molar concentration. For each protein and spectral feature, we offset the response relative to the mean of its triplicate at the lowest tested concentration (0.015 µg/mL), so that the lowest concentration defined the zero baseline. All subsequent steps used this relative response.

To match the accessible concentration range of our own titration, which extended to 1 µM, the analysis window for each protein was capped at the concentration nearest 1 µM in log space; that concentration and all lower concentrations were retained, and higher concentrations were excluded. Because molecular weights differ among proteins, five to seven concentrations were retained for each protein.

Each protein-feature response was classified on the retained window. A response was called linear if Spearman's rank correlation between concentration and relative response was significant ($p < 0.05$); the sign of the correlation gave the direction. A response that was not linear was called near-baseline if the relative responses at the highest retained concentration differed from those at the lower retained concentrations by Welch's t-test ($p < 0.05$) and a concordant monotonic trend was confirmed by the Jonckheere-Terpstra test in the direction of the top-concentration change. This monotonicity requirement excluded single-concentration deviations that the t-test alone would admit. A called response was additionally required to exceed a magnitude floor at the highest retained concentration, set at 0.1 nm for the wavelength features and 5% for the intensity features. Responses below the cutoffs were treated as not significant. Responses meeting neither the linear nor the near-baseline criterion were classified as silent. For each response, a three-parameter Hill model was fit to the retained points by nonlinear least squares as described in our Methods section.

Supplementary Note 1: Residue-level contrasts between DNA and PEG coronas

Each amino acid is represented by three composition features, defined in **Table S2**. Seven of the twenty amino acids differ significantly between DNA and PEG coronas under both the Welch and Mann-Whitney tests, and three further residues differ under the Mann-Whitney test alone (see the table below).

Variable	p-value	Hodges-Lehmann Difference	DNA Median	PEG Median
% C	3.18E-05	1.25E-02	2.58E-02	1.27E-02
% Exp/Exp P	3.18E-05	1.16E-02	6.97E-02	6.03E-02
% Exp/All P	1.11E-04	9.80E-03	5.31E-02	4.43E-02
% P	1.11E-04	1.21E-02	5.97E-02	4.90E-02
% Exp/Exp R	4.76E-04	7.76E-03	7.72E-02	7.33E-02
% N	7.14E-04	2.66E-03	3.65E-02	3.41E-02
% Exp/All H	2.16E-03	3.41E-03	1.61E-02	1.29E-02
% Exp/Exp H	2.16E-03	3.82E-03	2.19E-02	1.74E-02
% H	2.16E-03	3.17E-03	2.03E-02	1.72E-02
% Exp/All E	5.49E-03	-9.11E-03	6.27E-02	6.98E-02
% E	1.59E-02	-9.63E-03	6.66E-02	7.51E-02
% Exp/All N	1.59E-02	1.95E-03	3.13E-02	2.96E-02
% Exp/Exp E	2.01E-02	-1.23E-02	8.74E-02	9.96E-02
% R	2.01E-02	4.49E-03	6.07E-02	5.72E-02
% D	3.14E-02	-2.57E-03	4.76E-02	5.01E-02
% Exp/All D	3.14E-02	-3.47E-03	3.98E-02	4.33E-02
% Exp/Exp T	3.87E-02	4.95E-03	5.96E-02	5.49E-02
% I	3.87E-02	-2.24E-03	4.31E-02	4.51E-02
% Q	3.87E-02	-4.16E-03	4.18E-02	4.51E-02
% Exp/All R	4.73E-02	4.19E-03	5.46E-02	5.12E-02
% Exp/All T	4.73E-02	3.20E-03	4.41E-02	4.03E-02

Cysteine, proline, histidine, asparagine, and arginine are enriched in DNA coronas, and glutamate and glutamine are enriched in PEG coronas under both tests, with exposed threonine, isoleucine, and aspartate reaching significance under the Mann-Whitney test only. These residues sort along two protein properties, helix propensity and charge:

1. Ranking residues by the direction and size of their DNA versus PEG difference follows helix propensity. Residues that disfavor alpha helix are enriched in DNA coronas and residues that favor it are enriched in PEG coronas. Across the fifteen uncharged residues, the Spearman correlation between the weighted DNA versus PEG difference and the helix propensity scale[2] is rho = 0.76 with p = 0.001. The correlation is unchanged when the difference is normalized for residue abundance, when proline is excluded as the extreme value of the scale, and when the independent Chou-Fasman scale is used[3], so it is not specific to one scale or driven by residue abundance (**Figure S12**). This axis sorts proline, cysteine, and asparagine, all enriched in DNA coronas, from glutamine and isoleucine, enriched in PEG coronas. It agrees with the macro-level observation in **Fig 3b** of the main text that DNA coronas carry less helix and more coil, and thus the residue composition

and the predicted secondary structure are two independent measurements of the same preference.

2. The second axis is charge. While both classes demonstrate enrichment in positively charged residues relative to plasma, only PEG shows an affinity for negatively charged residues (**Fig 3a**). Consistently, negatively charged residues are not significantly enriched in DNA coronas relative to PEG coronas, and protein pI correlates positively with DNA/PEG. We attribute this difference to electrostatic interactions. The negatively charged phosphate backbone of the DNA wrapping attracts cationic protein surfaces and repels negatively charged residues. Conversely, because PEG surfaces are largely neutral, binding is driven by hydrophilic interactions. This would explain why PEG surfaces attract positively and negatively charged residue-containing proteins equally, and why exposed neutral polar residues prefer PEG over DNA.

We also find that histidine carries the aromatic difference. Exposed aromatic content is significantly higher in DNA coronas (**Fig 3b** in the main text), and that class comprises phenylalanine, tyrosine, tryptophan, and histidine. While the three neutral aromatics are null, only histidine (both %H and % Exp H) is significant. Histidine is the only aromatic residue that is ionizable near neutral pH, with a side-chain pKa near 6.5 in proteins, and it is also a poor helix former. Thus, both electrostatics and helix propensity predict its enrichment in DNA.

Regarding cysteine, %C is strongly enriched in DNA coronas while its exposed fraction shows no difference and is slightly more enriched in PEG (% Exp/All C). The cysteine signal therefore comes from buried cysteine, which in plasma proteins is predominantly disulfide-bonded cysteine. This suggests that DNA wrappings prefer disulfide-stabilized, conformationally restrained proteins. The higher proline fraction in the DNA group can also be explained by rigidity. Proline restricts backbone conformation and is the strongest helix breaker.

Regarding arginine, its enrichment is the one charge-related result that the two factors above do not fully account for. Arginine and lysine carry the same formal charge and have nearly identical helix propensities. However, only arginine is significantly enriched in DNA coronas. The separation between the two residues likely arises from differences in side-chain chemistry. The guanidinium group of arginine can form bidentate hydrogen bonds with the phosphate backbone and stack on DNA-SWCNT sidewall, whereas the ammonium group of lysine forms a single point contact, and arginine is correspondingly the residue most over-represented at protein-DNA interfaces. We thus read the polymer difference as conformational selection with a secondary contribution from contact chemistry at the arginine position.

Supplementary Note 2: Descriptor-resolved effects of QWD chemistry on corona composition

Five chemical descriptors of the aryl QWD were considered with the following physicochemical categories: electronic (Hammett constant), steric (hydrodynamic size), and thermodynamic (H-bond capacity, logP). To distinguish genuine descriptor-specific effects from collinearity artifacts, we focus below on features exclusively significant for one descriptor class.

The electronic character of QWDs correlates with the depletion of positively charged surface residues. More H-donating and hydrophobic QWDs negatively correlate with GRAVY and positively with exposed aromatics, indicating a preference for hydrophilic proteins with high surface exposed aromatics. At the amino acid level, electron-withdrawing QWDs uniquely enrich methionine and deplete lysine, indicating a preference for uncharged protein surfaces. H-bond accepting QWDs enrich strong H-bond donors, e.g., glutamine, while H-bond donor QWDs preferentially recruit acceptor-capable surface residues. Surface serine, phenylalanine, and tyrosine residues, which can accept hydrogen bonds or participate in hydrogen-pi interactions, positively correlate with this axis.

Structural features, including flexibility and secondary structure information, are significant for both Hammett and the thermodynamic axis, with opposite correlations. However, because Hammett and the thermodynamic axis are anti-correlated, these structural features cannot be attributed to either descriptor independently. Resolving this conformational selection trend would require an expanded library in future work.

In terms of steric space, bulkier QWDs recruit larger, more polar corona proteins, but does not significantly correlate with any structural or flexibility metric. This differentiates the steric axis related to protein size and polarity selection from the conformational effects captured by the electronic/thermodynamic gradients described in the main text.

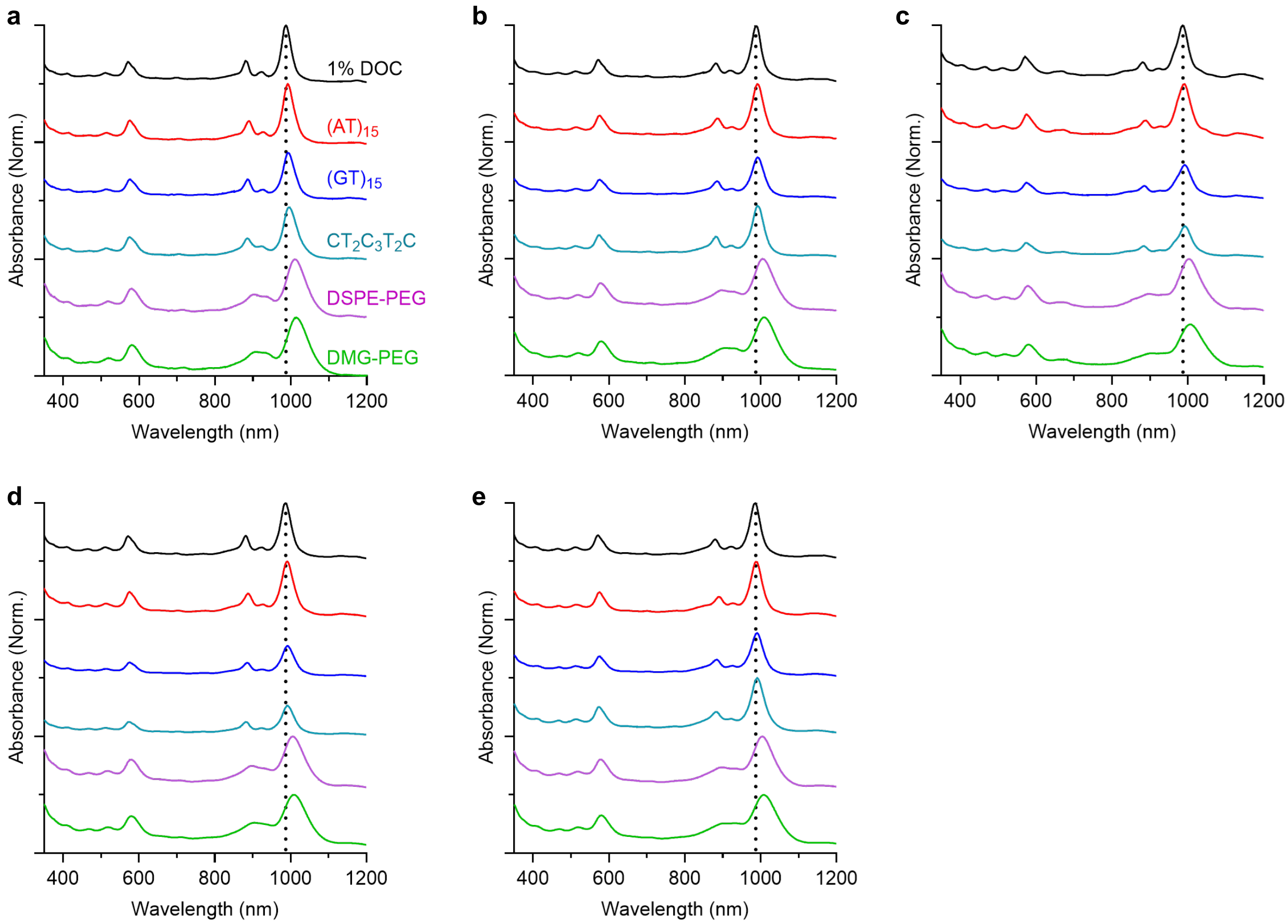

Figure S1. Absorbance spectra of chirality separated QWNT stocks before polymer exchange and after rewrapping with $(AT)_{15}$, $(GT)_{15}$, $CT_2C_3T_2C$, DSPE-PEG, DMG-PEG. Spectra are normalized to (6,5) E_{11} absorbance band. Dotted vertical line centered on (6,5) E_{11} center wavelength in 1% DOC (aq.) to visualize red shifting of polymer wrapped nanotubes. a, Pristine, b, Amino, c, Dinitro, d, Carboxyl, e, Trifluoro.

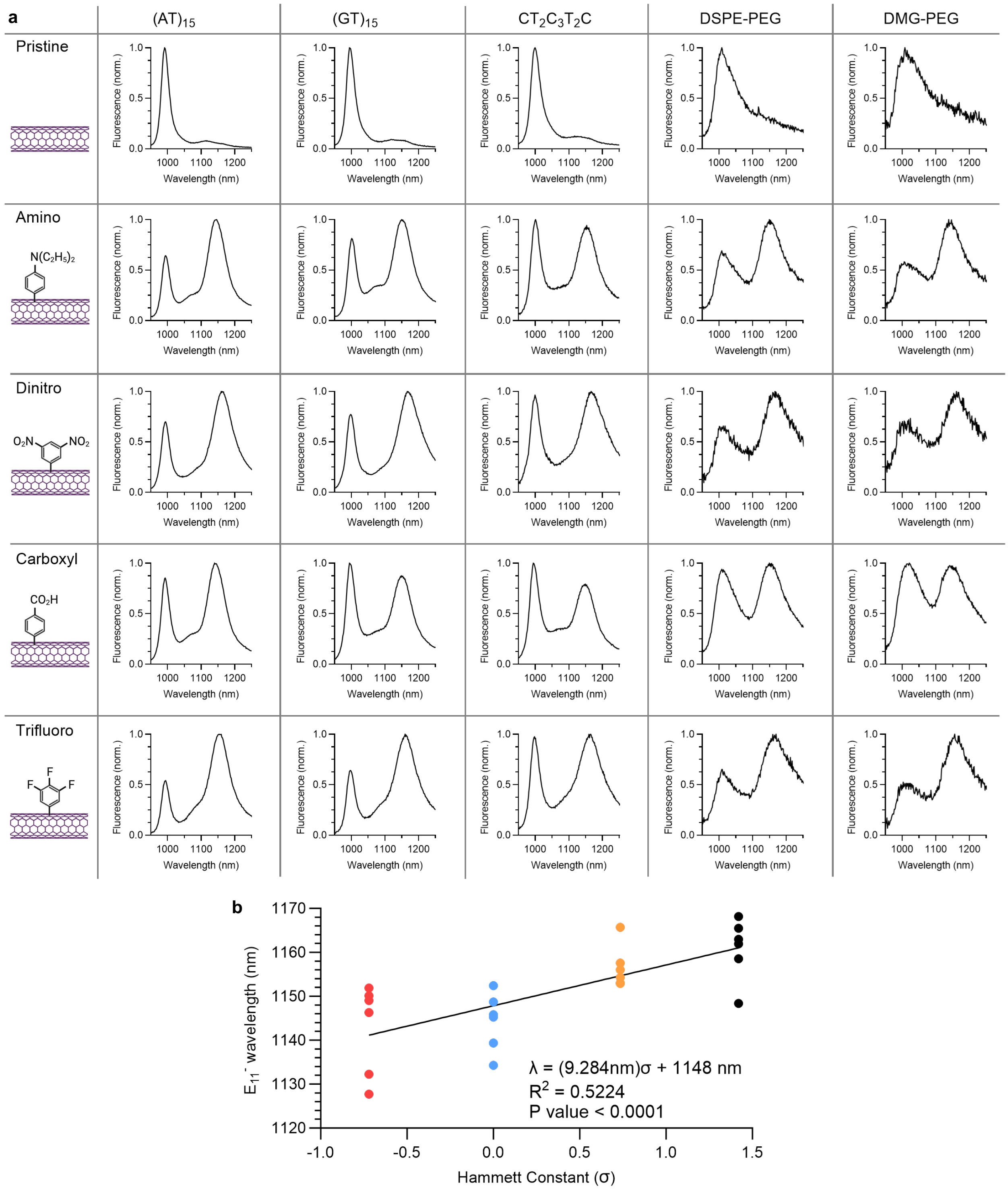

Figure S2. QWD emission of polymer-wrapped carbon nanotubes. **a,** Normalized fluorescence spectra of nanotube stock solutions in 1x PBS. The concentration of (6,5)-SWCNTs is 2 ug/mL. Rows indicate QWD identity; columns indicate polymer identity. **b,** E_{11}^{-} peak wavelength versus the Hammett constant of the aryl substituent of QWDs. Each point is one construct, measured in one of six surface environments (1% sodium deoxycholate and the five polymer wrappings of **a**). The line denotes the least-squares fit to all 24 constructs.

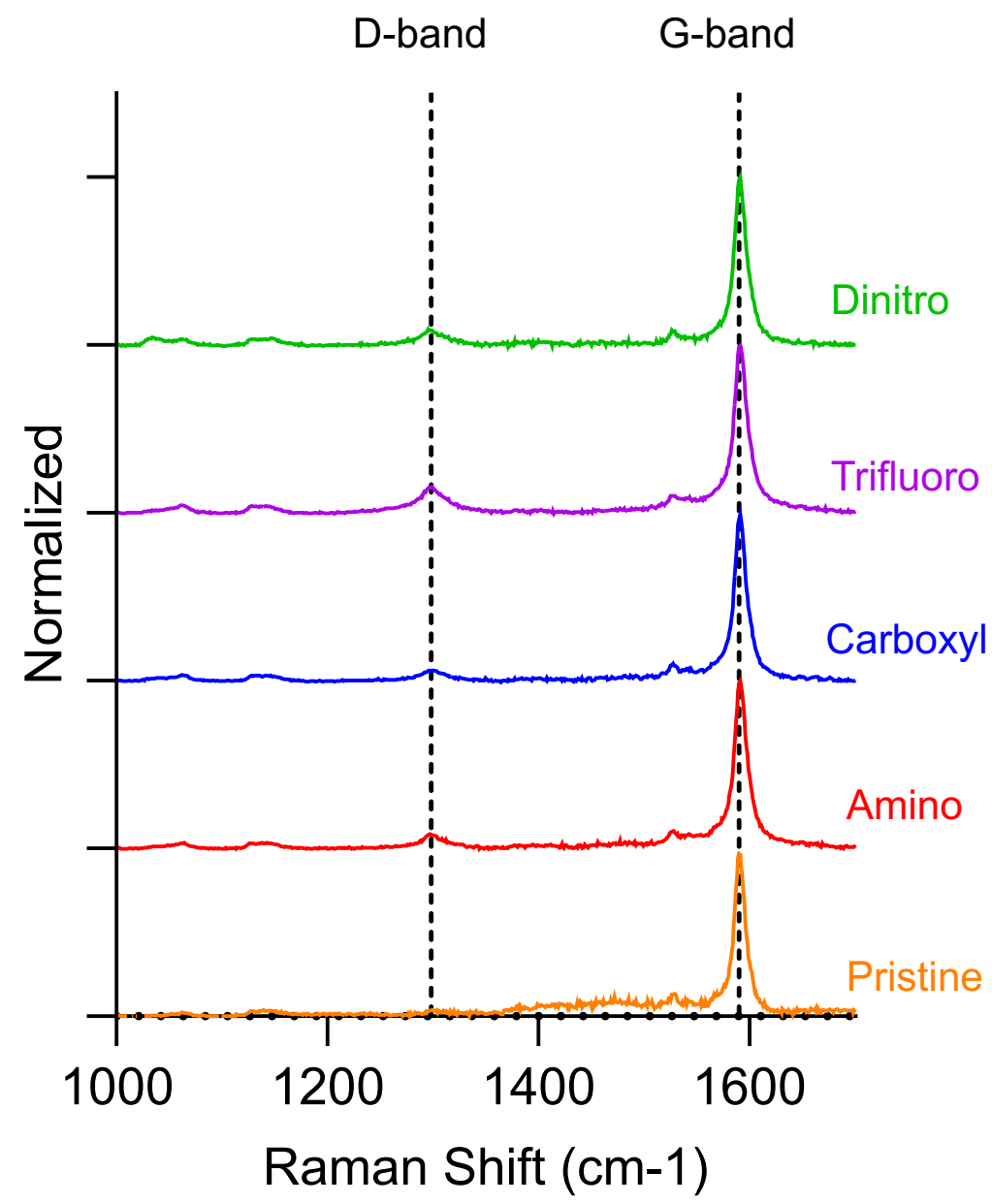

Figure S3. Raman spectra of pristine and QWD-functionalized nanotubes. Full Dashed lines indicate Raman D and G bands. Each spectrum was acquired with 40 mW of 785 nm laser excitation and normalized with respect to the G-band.

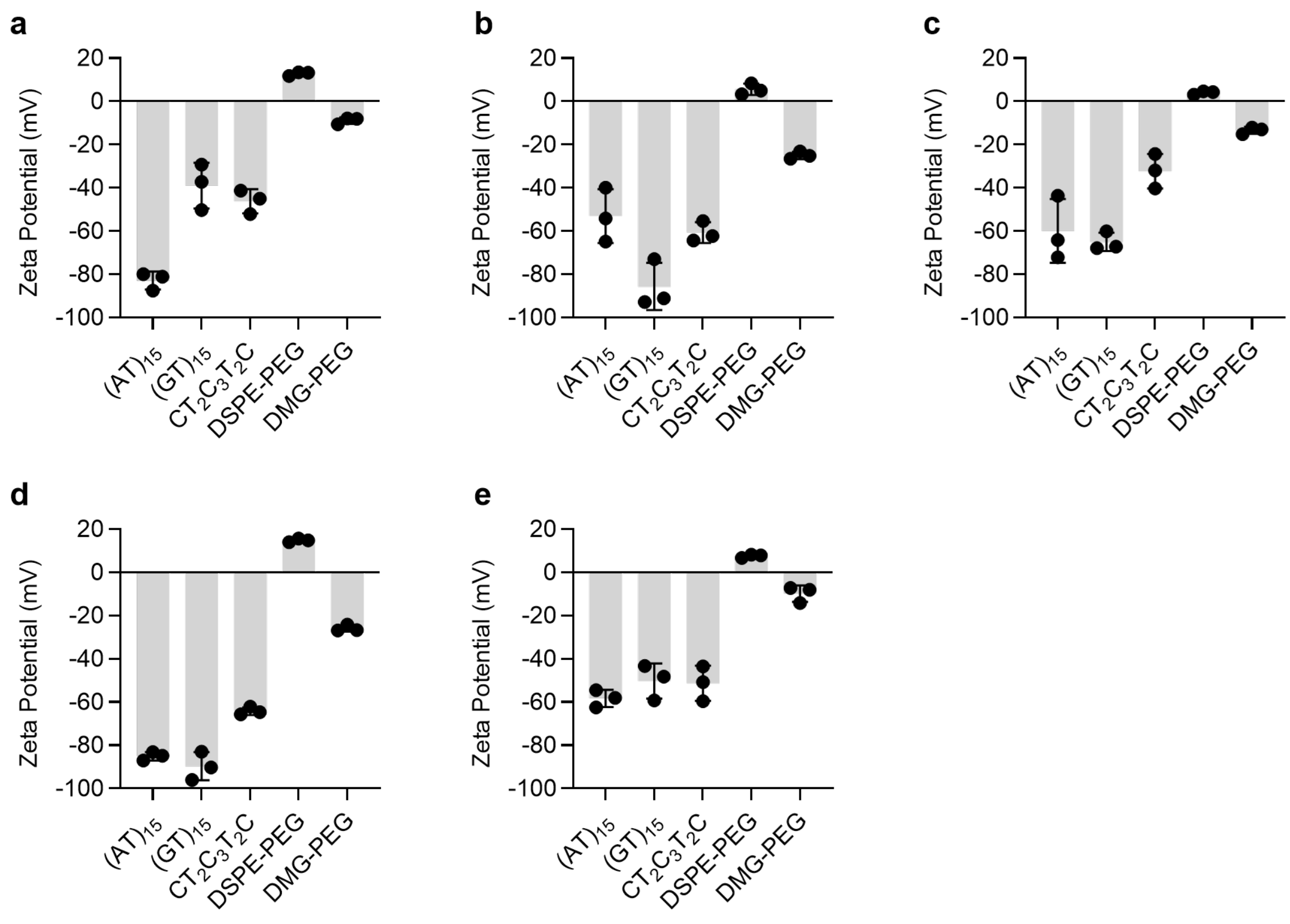

Figure S4. Zeta potential of polymer-wrapped QWNTs at 0.15 OD of (6,5) E_{11} in DI water. Error bars represent **the** standard deviation of triplicates. **a,** Pristine, **b,** Dinitro, **c,** Amino, **d,** Carboxyl, **e,** Trifluoro.

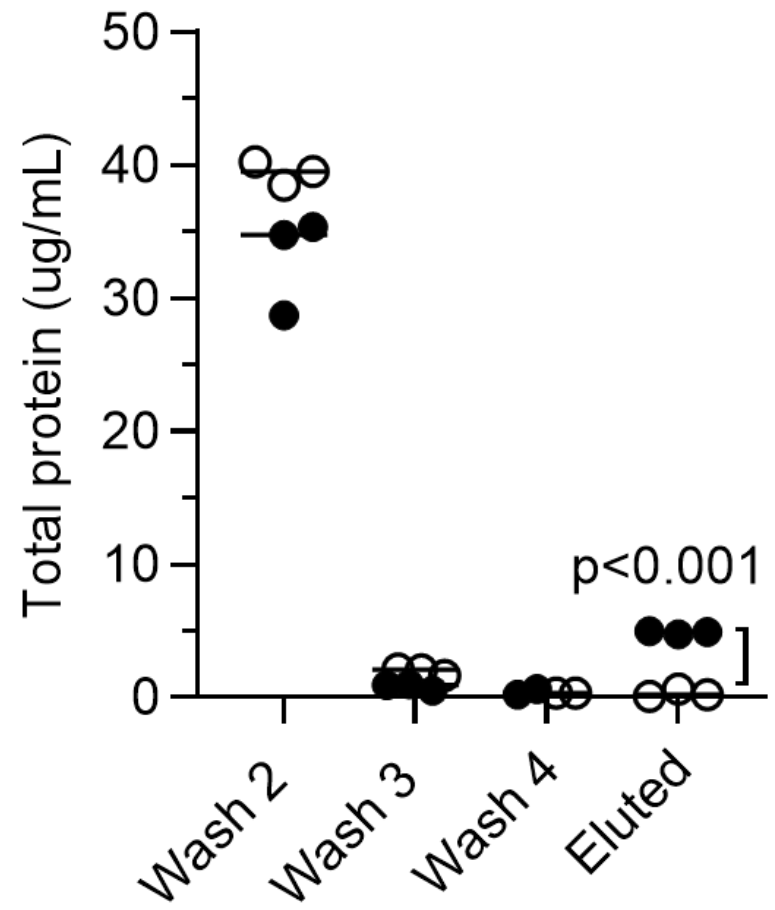

Figure S5. Bradford quantification of protein in the 1x PBS wash supernatants and in the eluate from the nanotube pellet. Filled circles are the SWCNT-included triplicates; empty circles are SWCNT-excluded controls, which were carried through the identical plasma incubation, washing, and elution procedure. Each point is an independent replicate (n = 3 per group). Residual plasma protein declines from 35 to 40 μg/mL at wash 2 to below 2 μg/mL at wash 3 and to the assay baseline at wash 4. The wash series is indistinguishable between the SWCNT-containing samples and the SWCNT-free controls, showing that the wash steps remove unbound plasma protein to the same extent. The two groups diverge only in the eluate, where the SWCNT-containing samples yield approximately 1 μg of protein while the SWCNT-free controls remain at baseline (unpaired two-tailed t-test, $p < 0.001$). Protein recovered in the eluate is therefore bound to the nanotube surface.

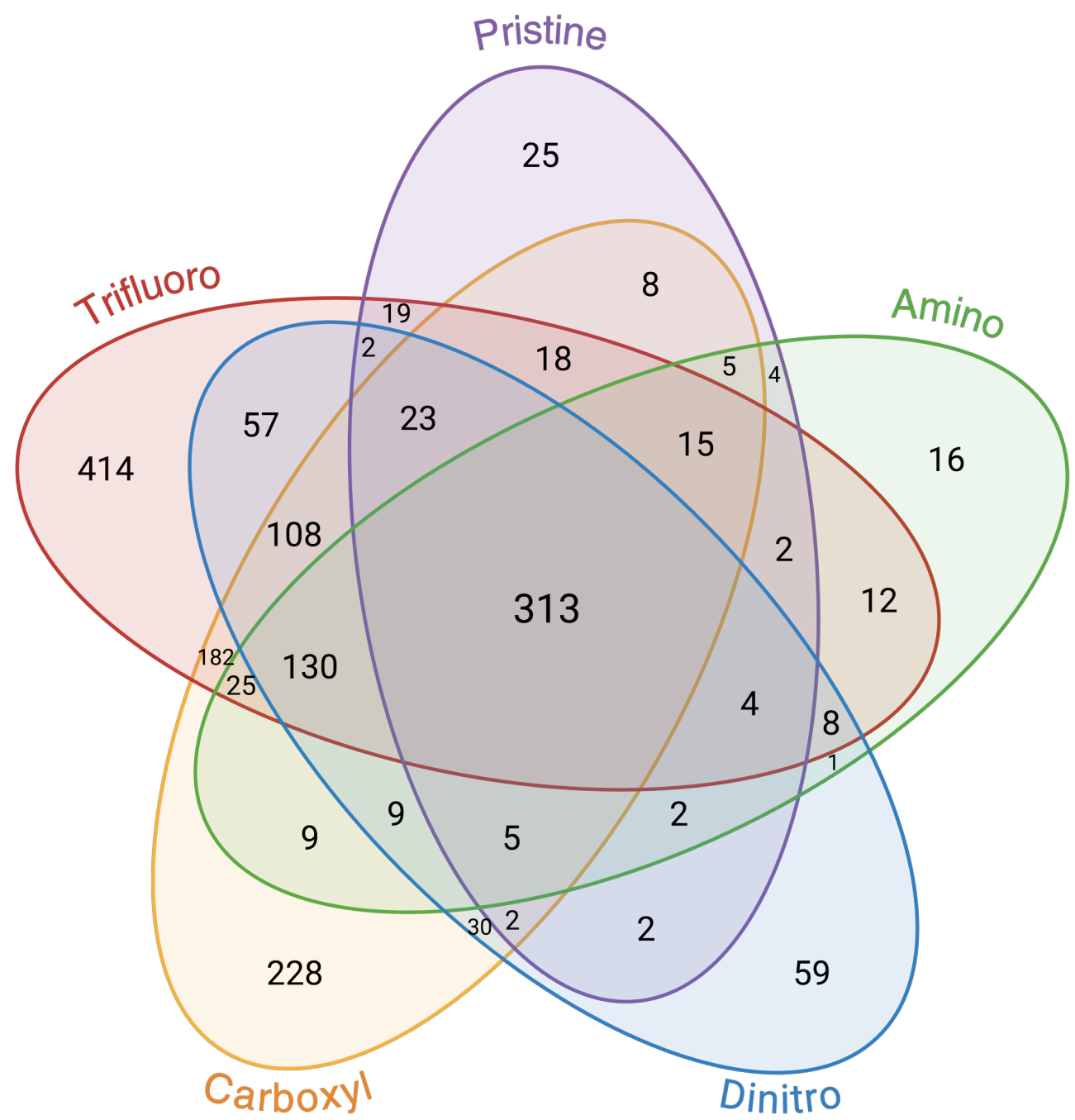

Figure S6. Five-way Venn diagram of proteins identified across the nanotube coronas. Values indicate the number of proteins shared among the indicated coronas.

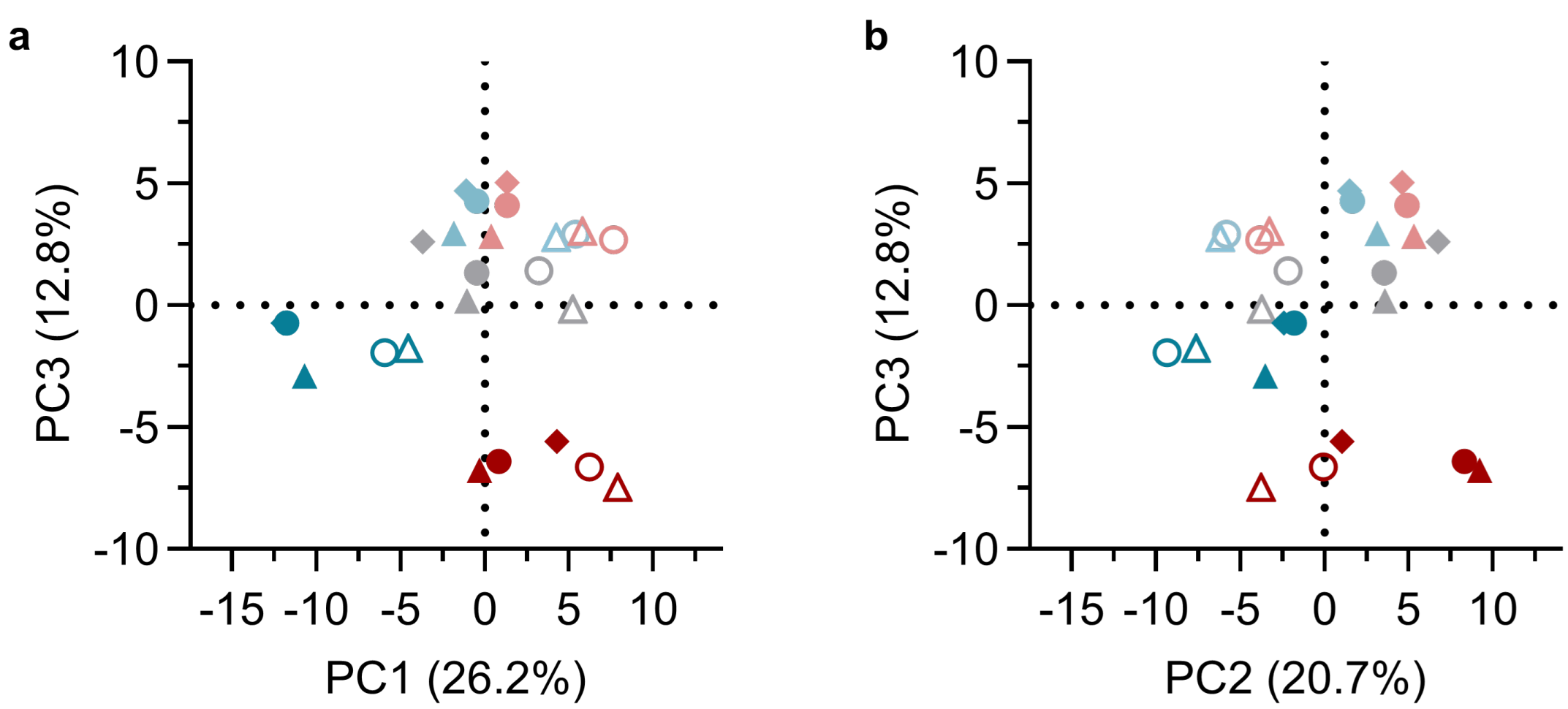

Figure S7. PCA plot of all sensor coronas, derived from Log2 protein fold-changes in 289 common proteins. Red, light red, gray, light blue, and blue symbols indicate dinitro, carboxyl, trifluoro, and aminoaryl QWNTs and pristine nanotubes, respectively. The filled triangle, circle, and diamond are $CT_2C_3T_2C$, $(GT)_{15}$, and $(AT)_{15}$ wrapping, respectively. The empty triangle and circle are DMG-PEG and DSPE-PEG wrapping, respectively. **a,** PC1 versus PC3. **b,** PC2 versus PC3.

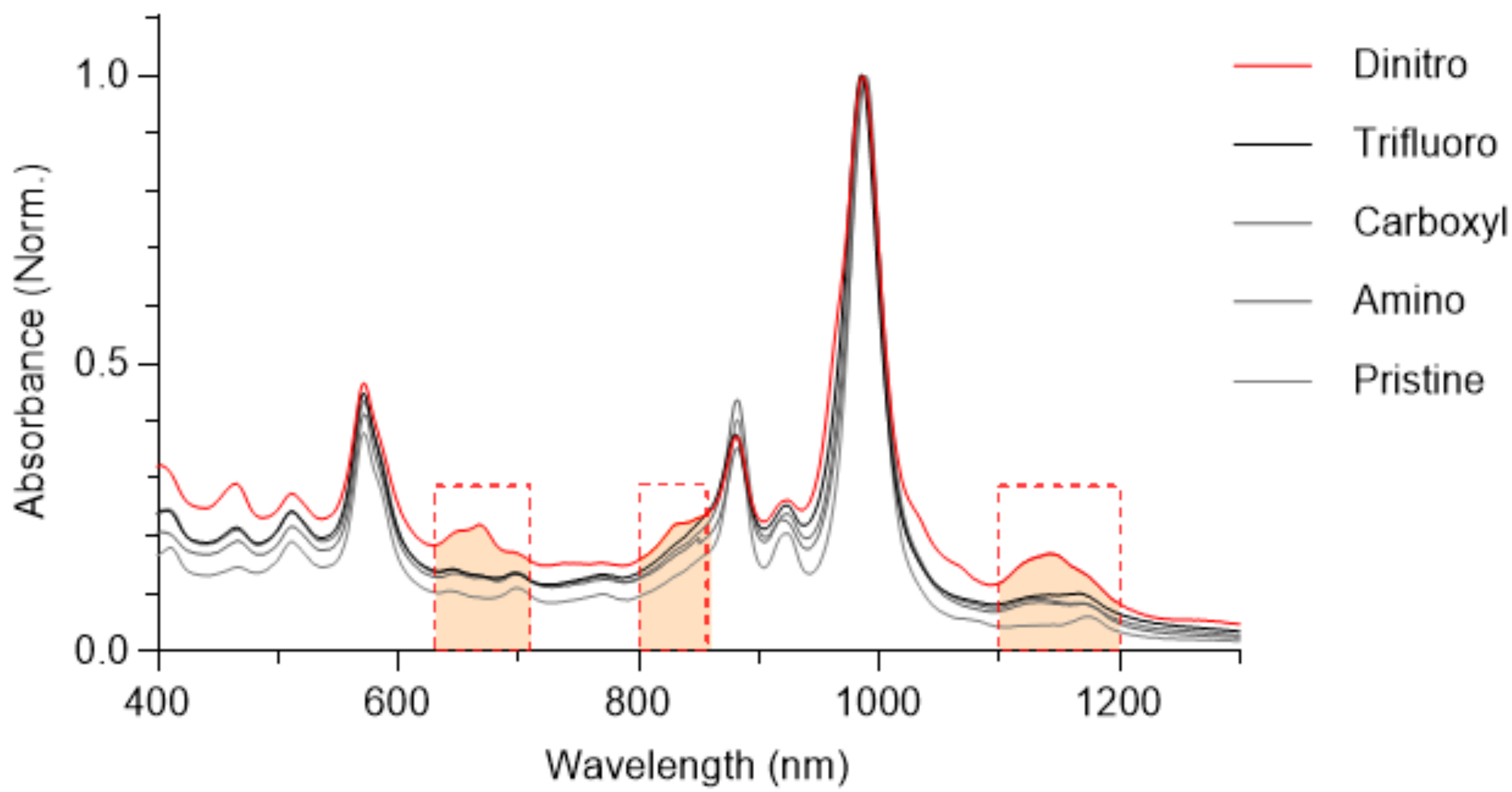

Figure S8. Normalized, overlaid absorbance spectra of nanotube stock in 1% DOC (aq.). Highlighted regions in dashed red boxes mark E_{11} and E_{22} absorption bands from non-(6,5) chiralities observed in 3,5-dinitroaryl QWNTs.

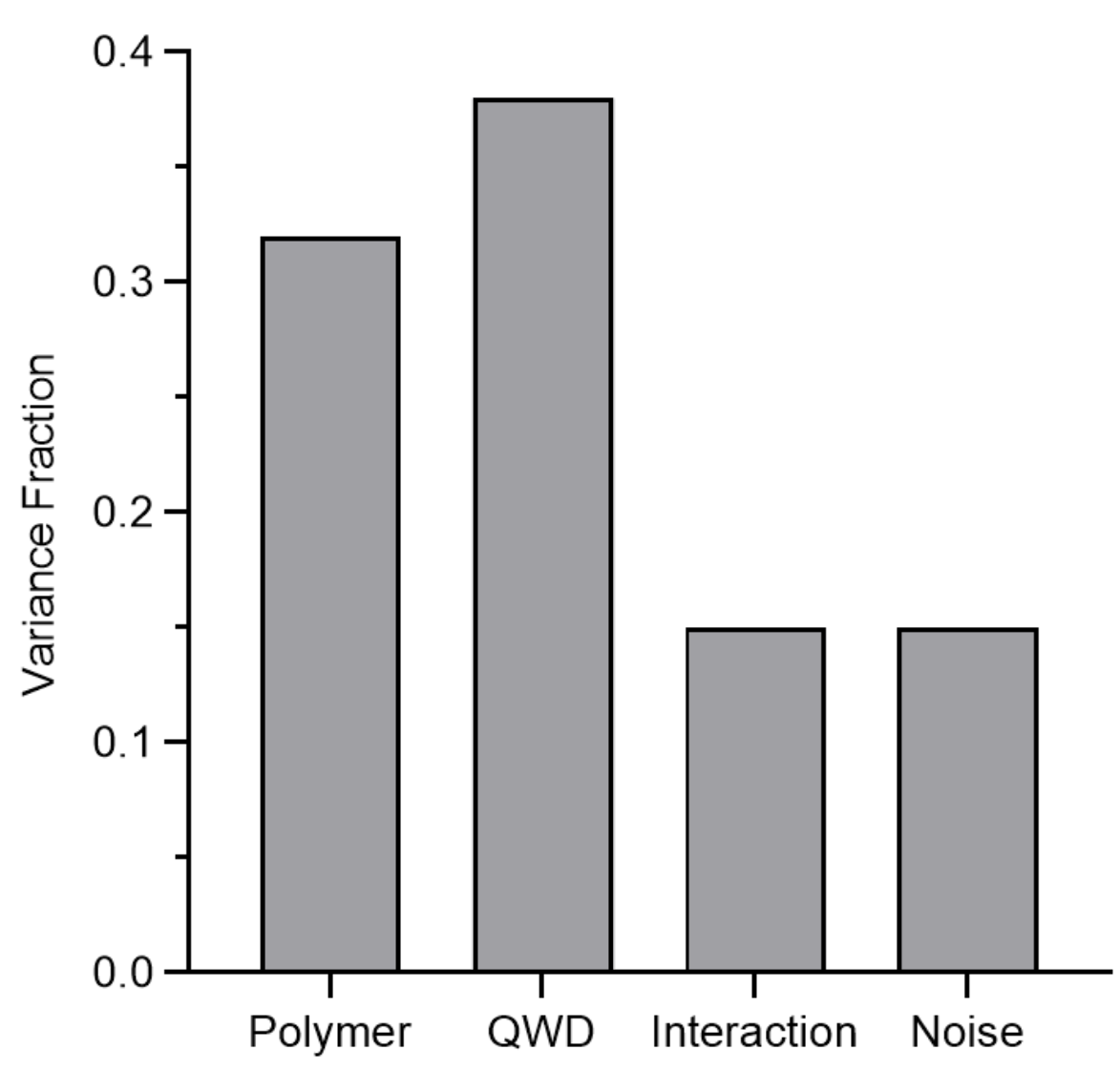

Figure S9. Partitioned variance in corona compositions for common proteins, excluding pristine coronas.

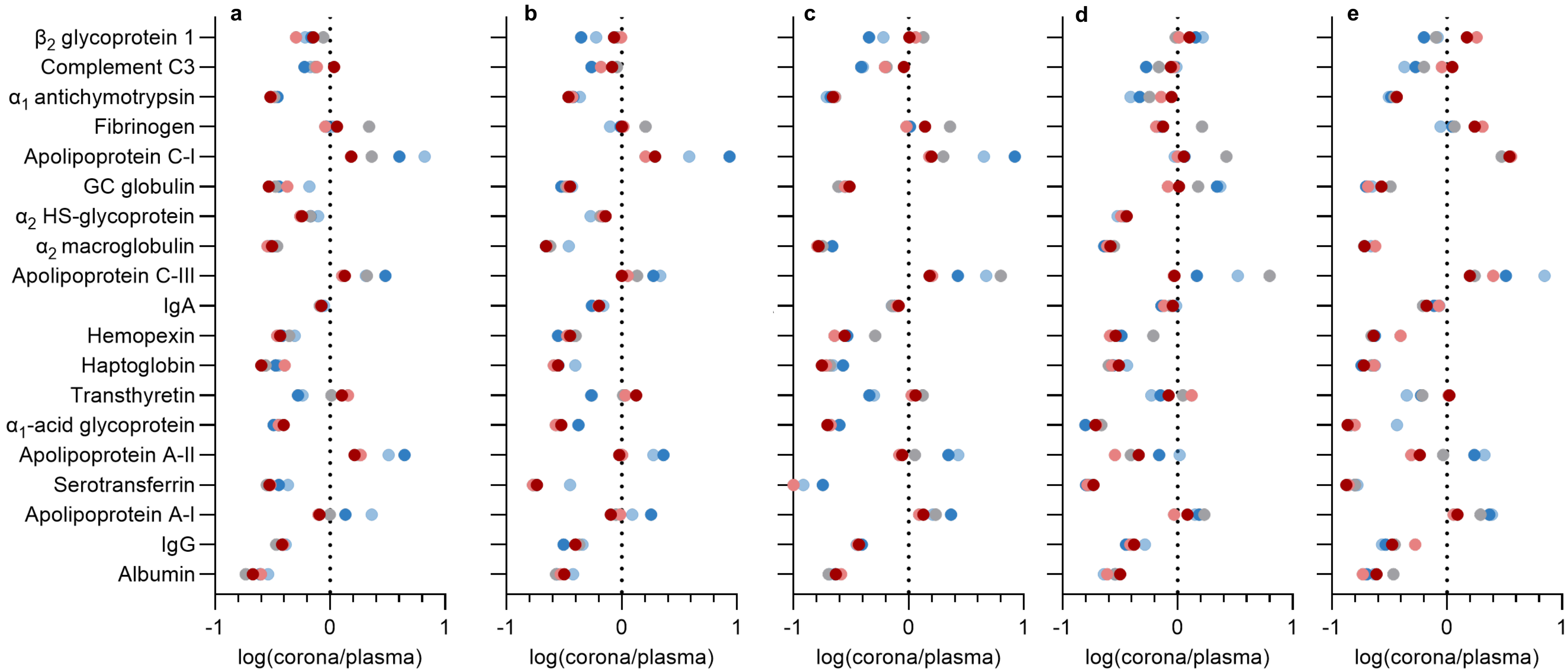

Figure S10. Log_2 fold-change plots of the 19 most abundant plasma proteins by molarity in each corona relative to plasma. **a,** Pristine, **b,** Amino, **c,** Dinitro, **d,** Trifluoro, **e,** Carboxyl. Blue: DSPE-PEG, Light blue: DMG-PEG, Gray: $CT_2C_3T_2C$, Light red: $(AT)_{15}$, Red: $(GT)_{15}$.

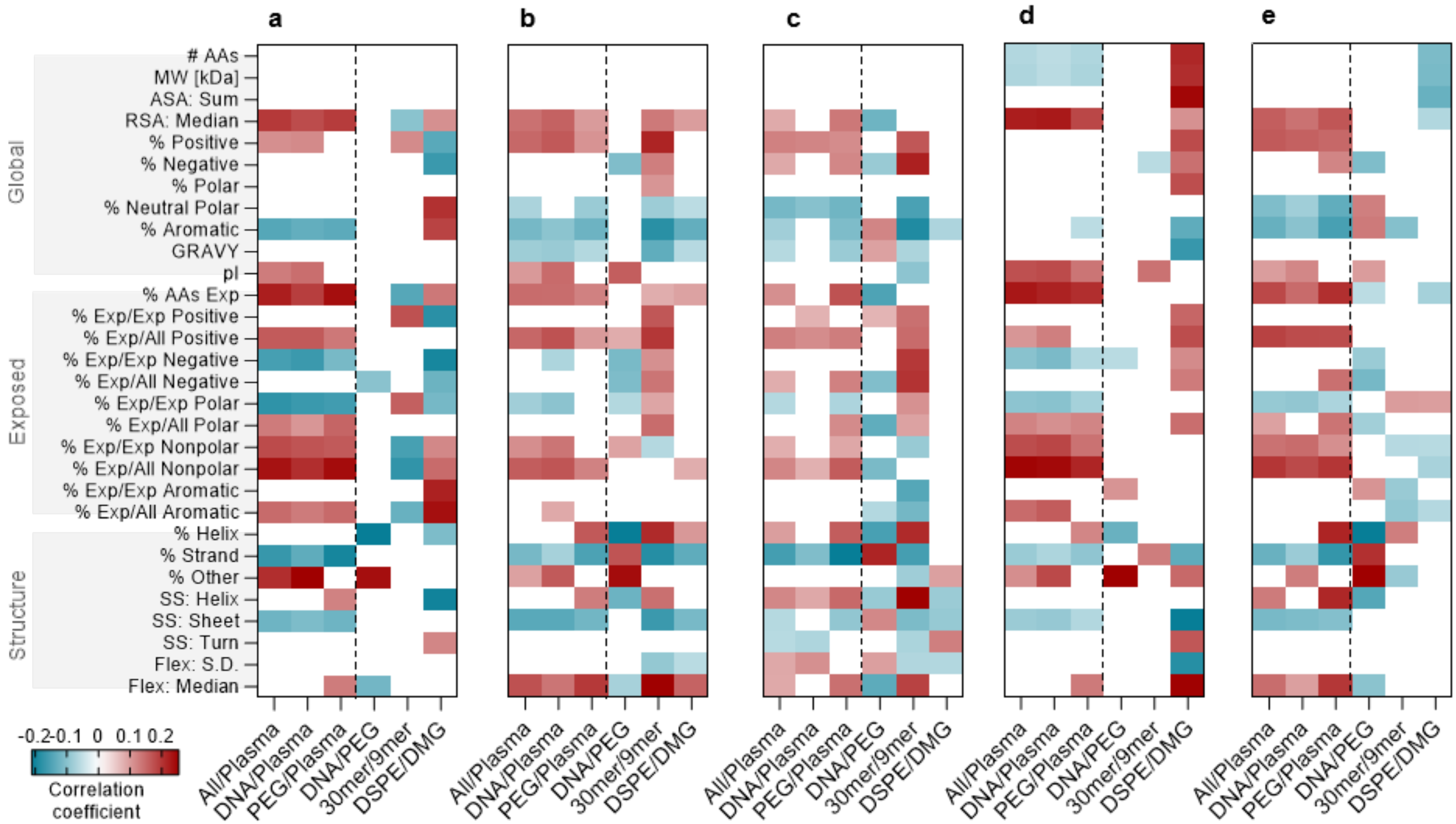

Figure S11. Heatmaps of Pearson rho values for correlations between protein features and corona fold change. **a,** Pristine, **b,** Amino, **c,** Dinitro, **d,** Trifluoro, **e,** Carboxyl.

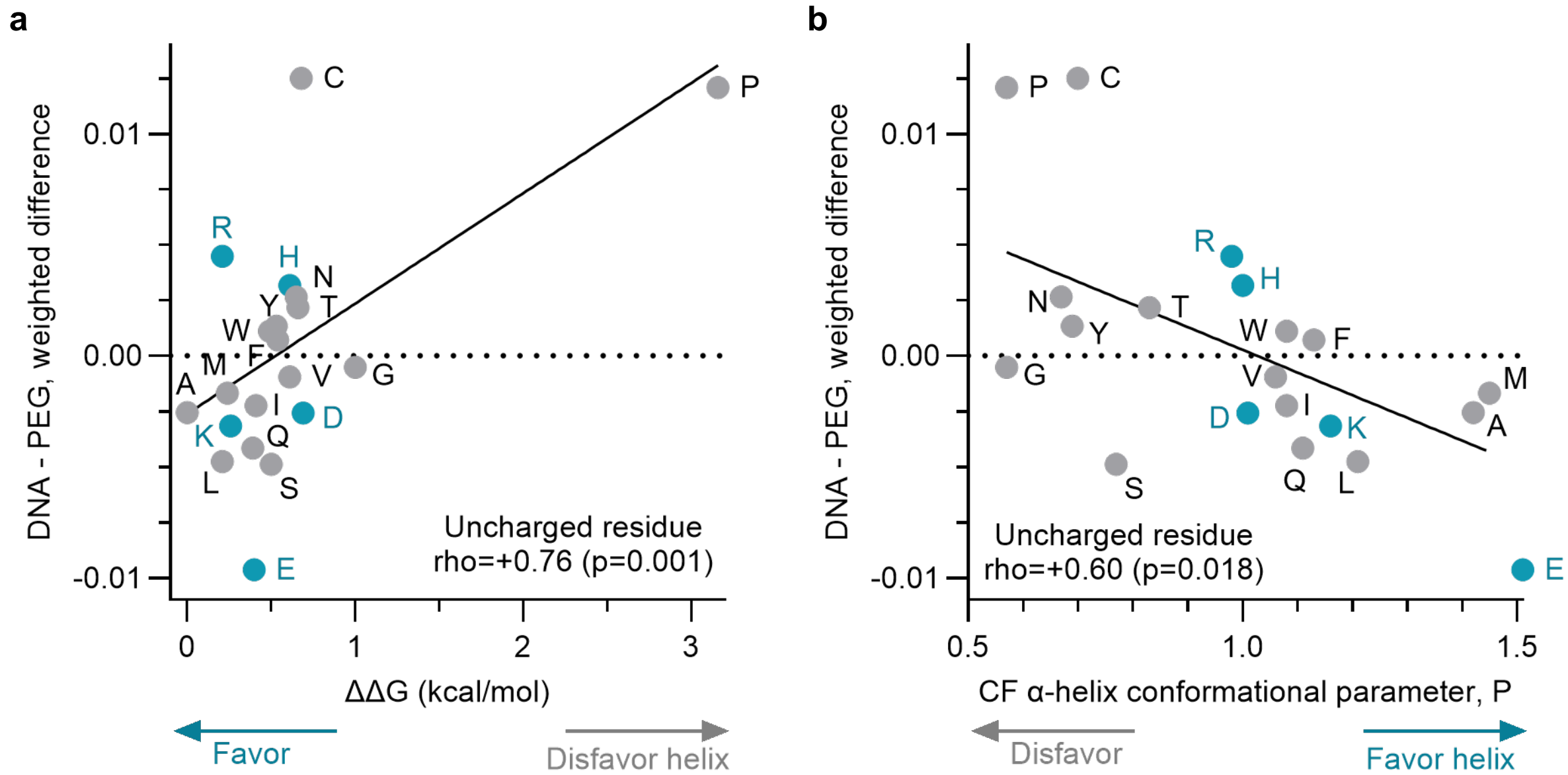

Figure S12. Per-residue DNA versus PEG difference tracks helix propensity. Correlation of the Hodges-Lehmann median difference between the DNA-wrapped and PEG-wrapped CNTs in the weighted amino acid residue and **a,** Pace-Scholtz helix propensity ΔΔG (ref 2) and **b,** Chou-Fasman alpha-helix conformation parameter (ref 3). Gray and blue points denote uncharged and charged residues at pH 7.4. Linear line is a least square fit to the uncharged residues.

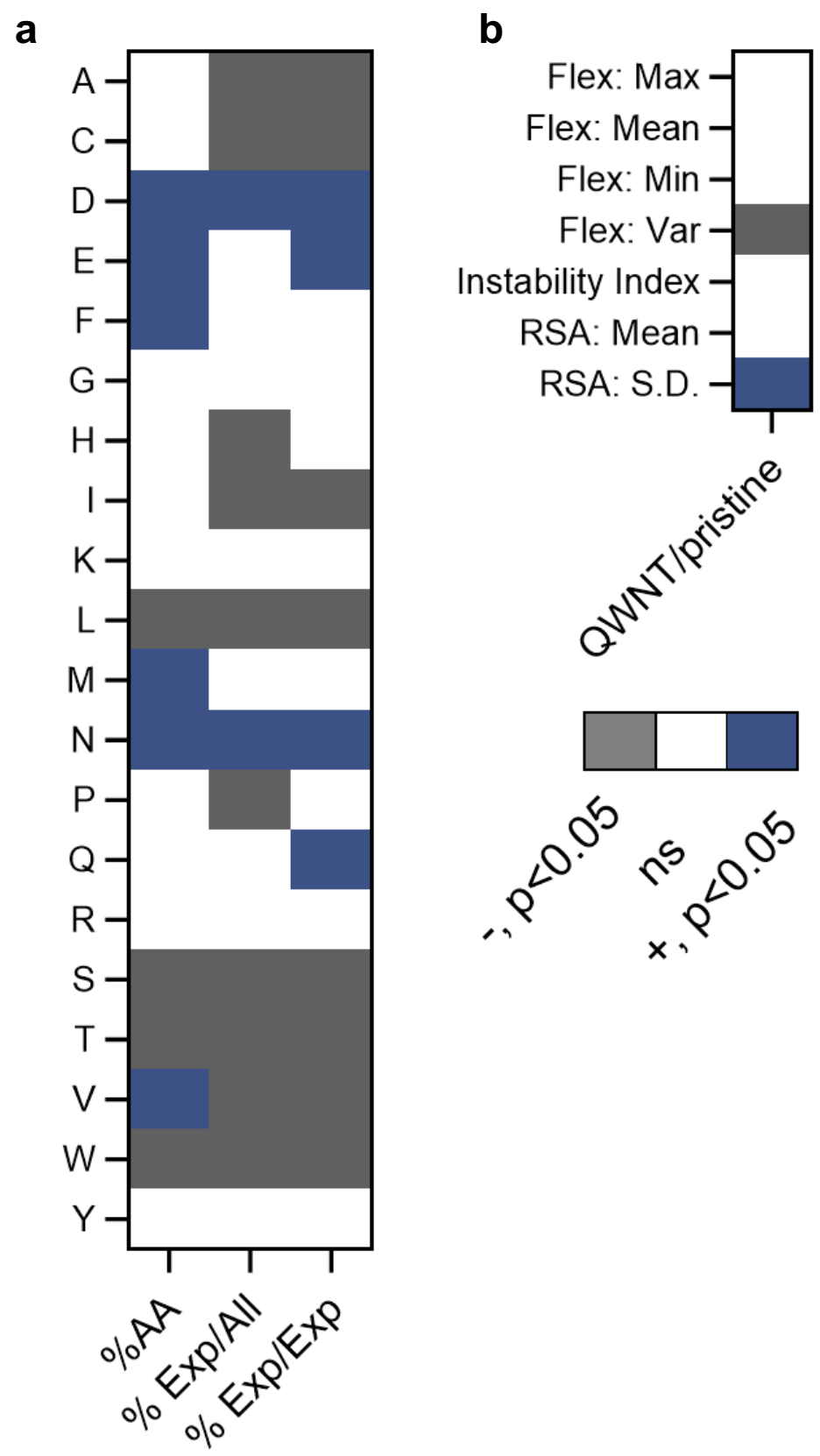

Figure S13. Heatmaps of Mann-Whitney test values comparing weighted protein features between QWNT and pristine nanotubes. **a,** Per-residue amino acid content, including %AA, % Exp/All, and % Exp/Exp. **b,** Flexibility, instability, and surface area features. Color indicates the direction of the significant ($p<0.05$) difference. Gray: Lower in QWNT; white: Not significant; Blue: Higher in QWNT.

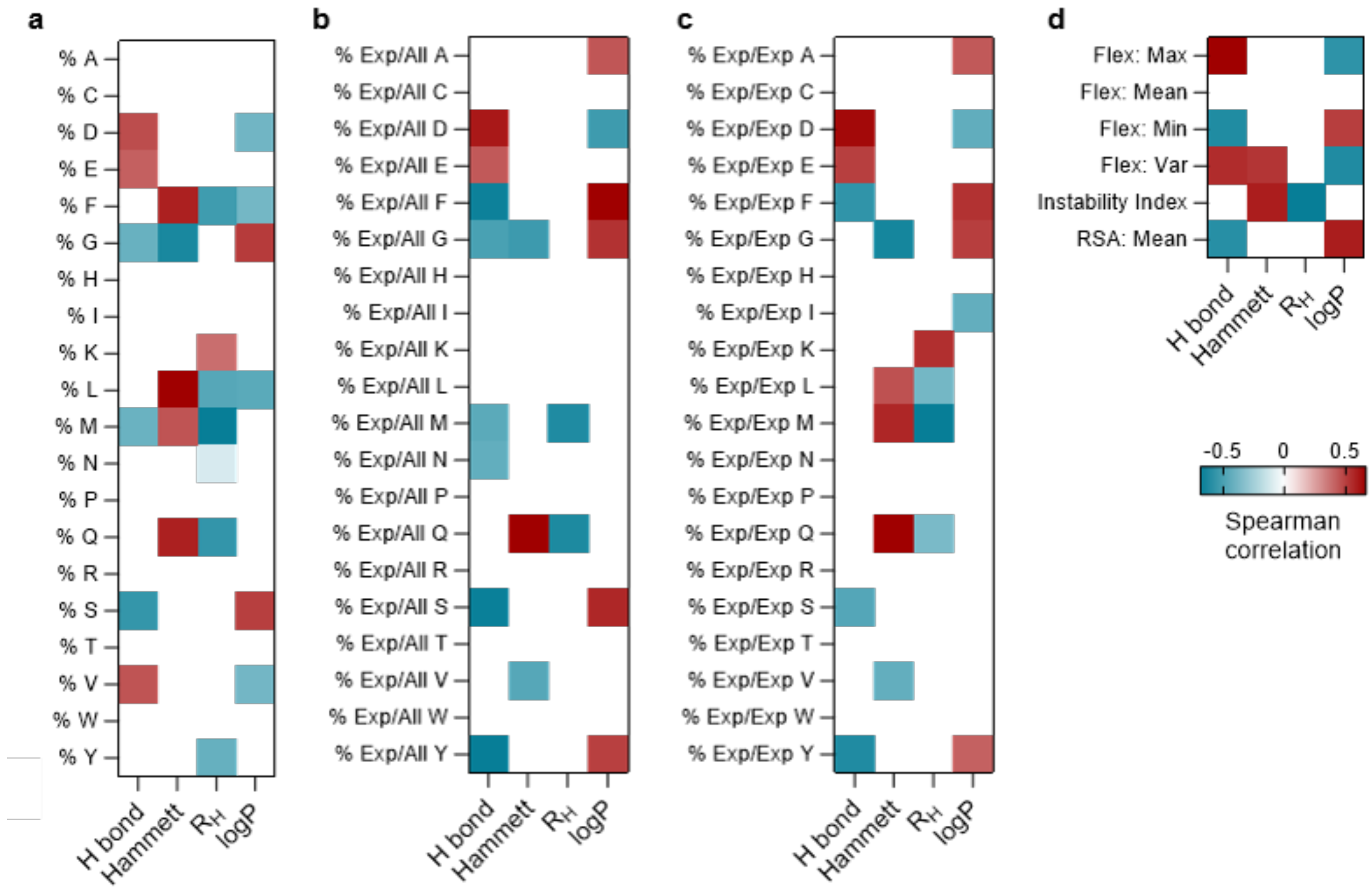

Figure S14. Heatmaps of Spearman rho values between weighted protein features and chemical parameters of QWD. **a,** %AA. **b,** % Exp/All. **c,** % Exp/Exp. **d,** Flexibility, instability, and surface area features. RSA: Relative surface area. Only significant correlations ($p<0.05$) are shown.

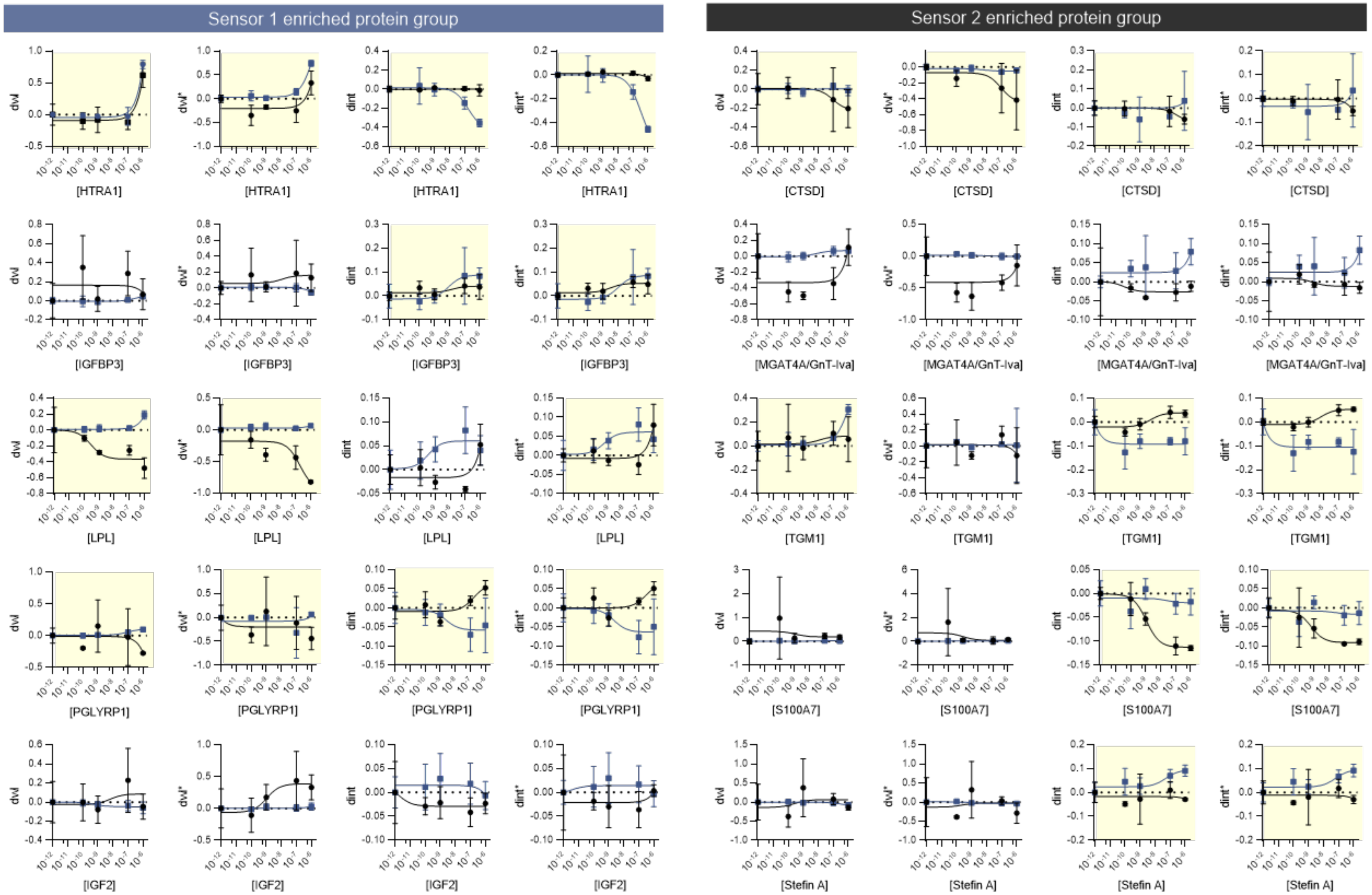

Figure S15. Sensor response in 20% plasma. Blue: Sensor 1 with Trifluoro QWD and $(AT)_{15}$ wrapping; Black: Sensor 2 with Amino QWD and DSPE-PEG wrapping. Yellow background marks a statistically significant spectral response. Curves are three-parameter Hill fits and guide the eye. Error bars give the standard deviation of technical replicates.

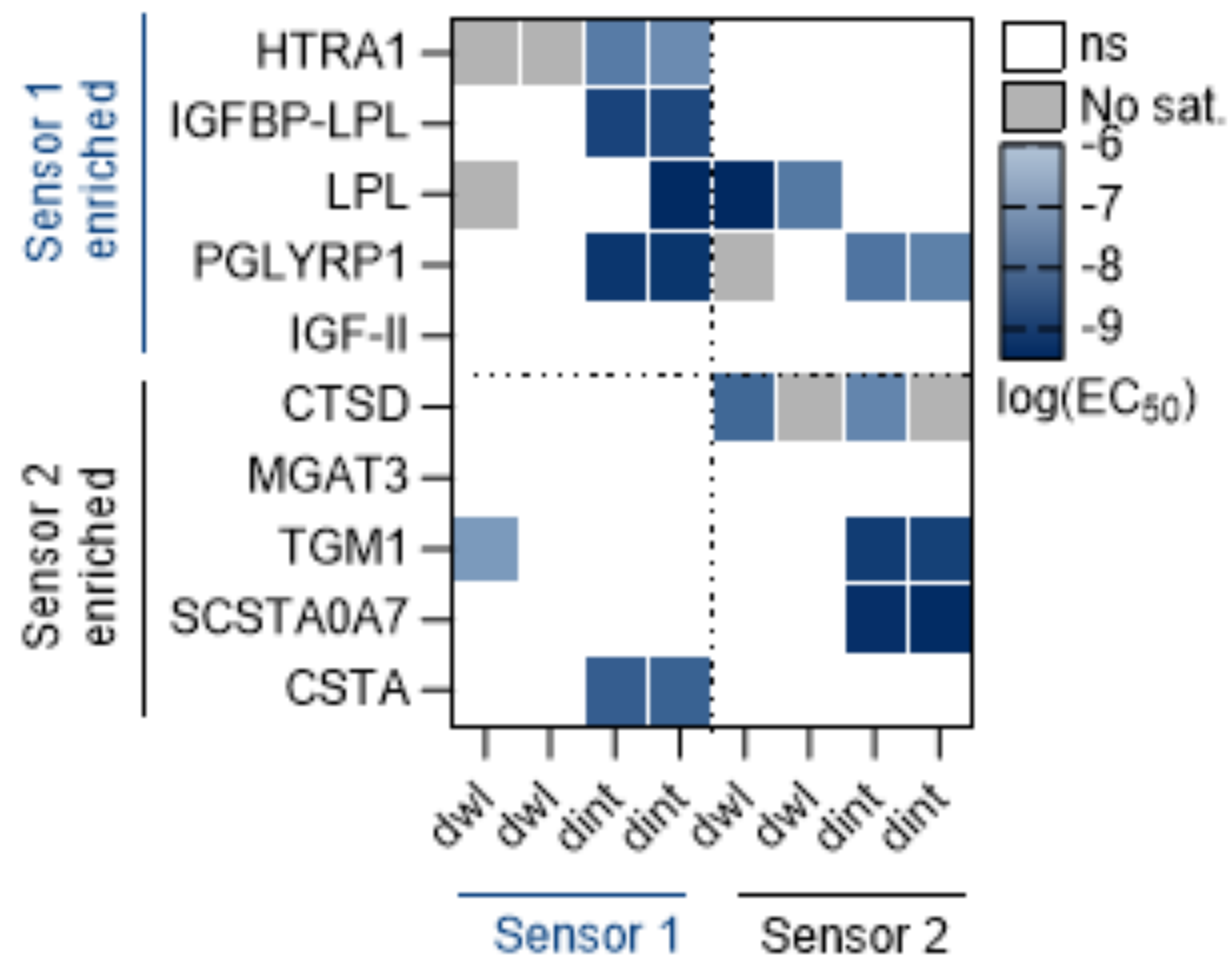

Figure S16. Heatmap of log-transformed EC_{50} values for sensor response across ten proteins and four spectral features per sensor. For active responses, apparent half-maximal response concentrations (EC_{50}) were estimated from three-parameter Hill fits. Color indicates log-transformed EC_{50} values in analyte concentration. Gray cells indicate the spectral responses did not saturate within the tested titration range and $EC_{50} > -6$.

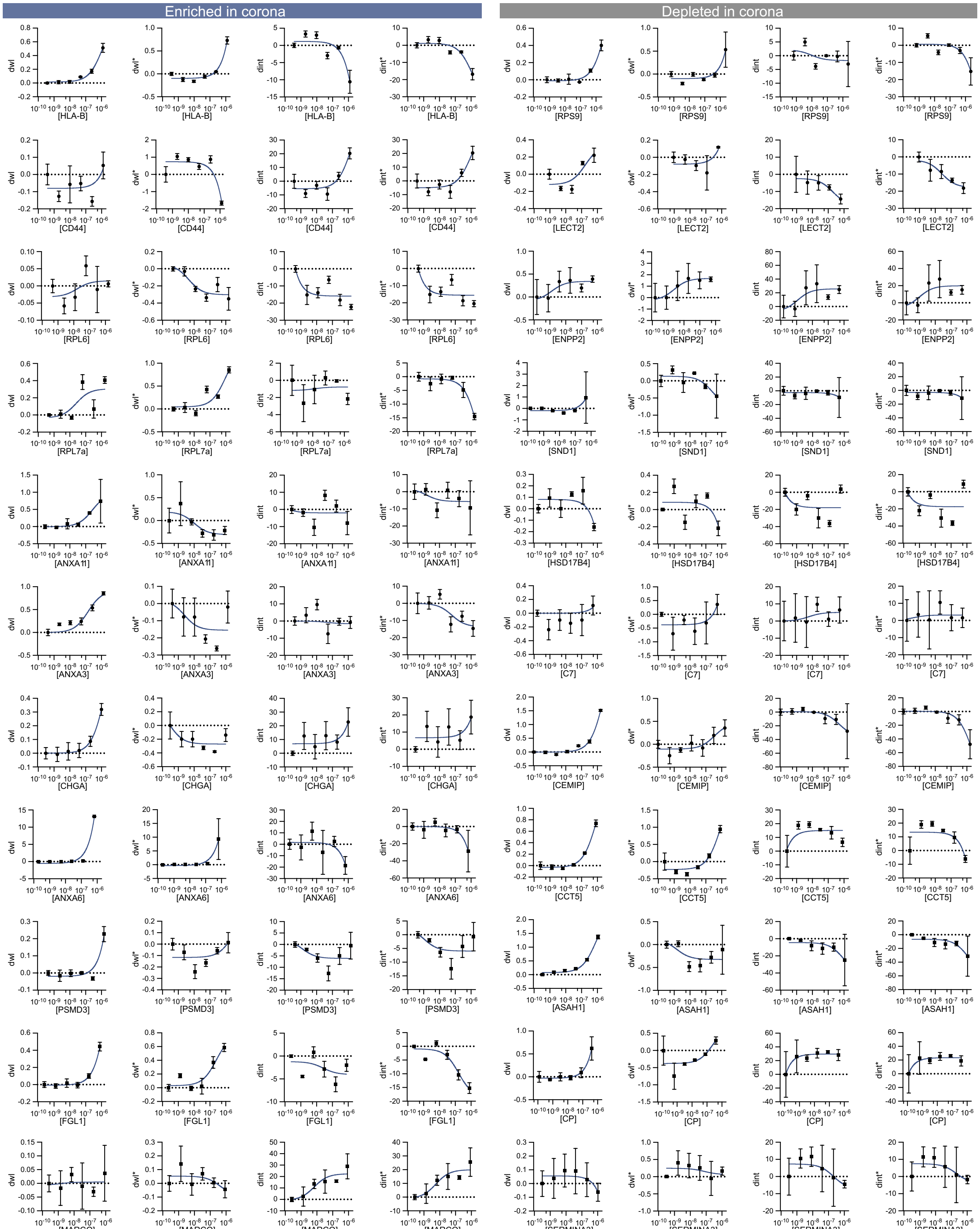

Figure S17. Fluorescence response of DSPE-PEG wrapped 3,4,5-trifluoroaryl QWNTs in 20% plasma. Fluorescence data were from the previous report by Goerzen et al.[1] Curves are three-parameter Hill fits and guide the eye. Error bars: Standard deviation.

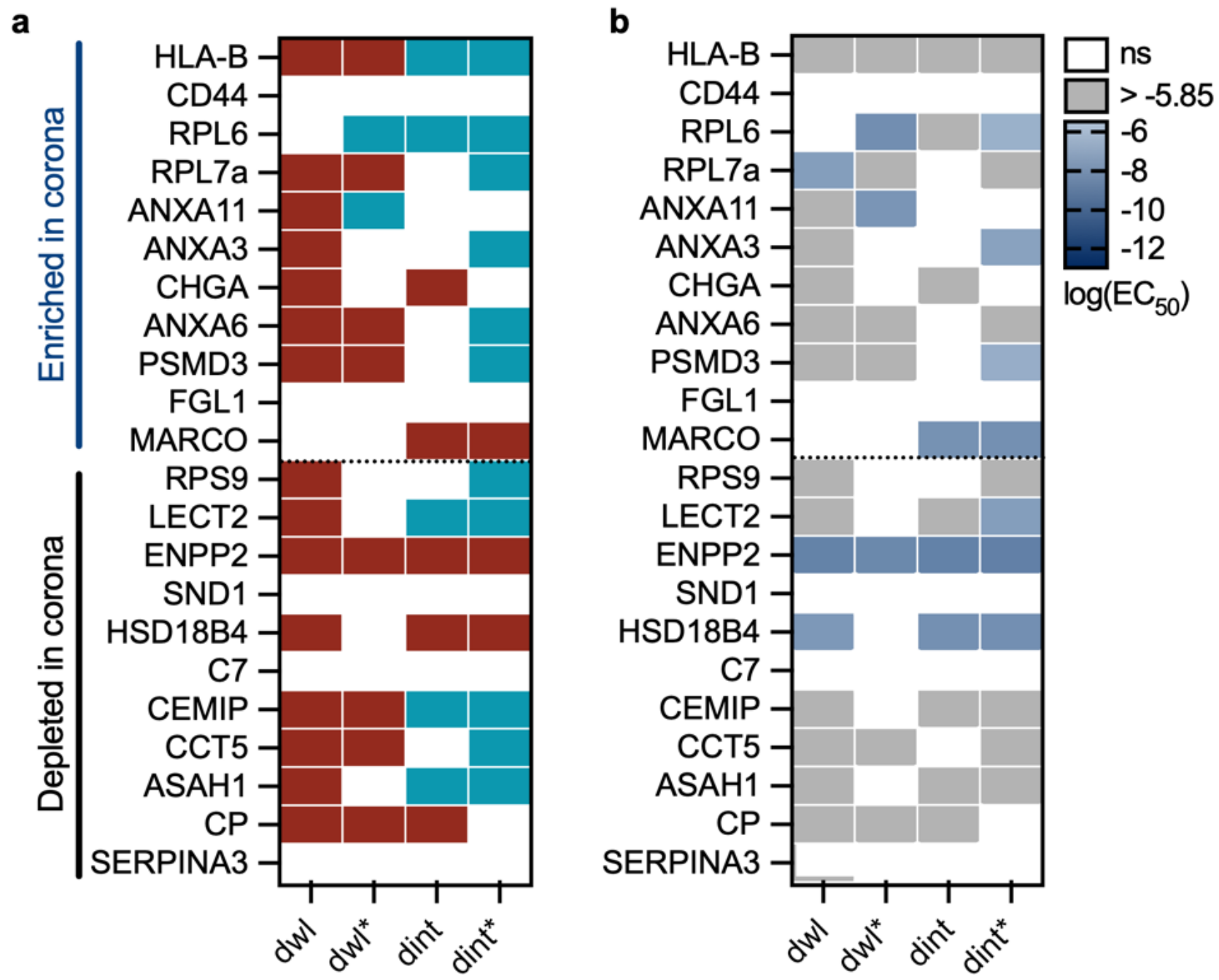

Figure S18. Heatmap of sensor response across an independent protein panel. Reanalysis of the fluorescence titration data of Goerzen et al. for DSPE-PEG wrapped 3,4,5-trifluoroaryl QWNTs, for the 22 proteins shared with our corona proteomics. Proteins are grouped as enriched (top) or depleted (bottom) in the corona. Responses were called from triplicate titrations over the ≤1 μM window. **a,** Direction of the significant responses. Red and blue cells indicate an increase and decrease of significant spectral response. White: not significant. The nanotube responded to enriched and depleted proteins at comparable rates (9/11 vs. 8/11). **b,** Half-maximal response concentration from three-parameter Hill fits. Color indicates log-transformed EC_{50} values in analyte concentration. Gray cells indicate the spectral responses did not saturate within the tested titration range and EC_{50} > 6.

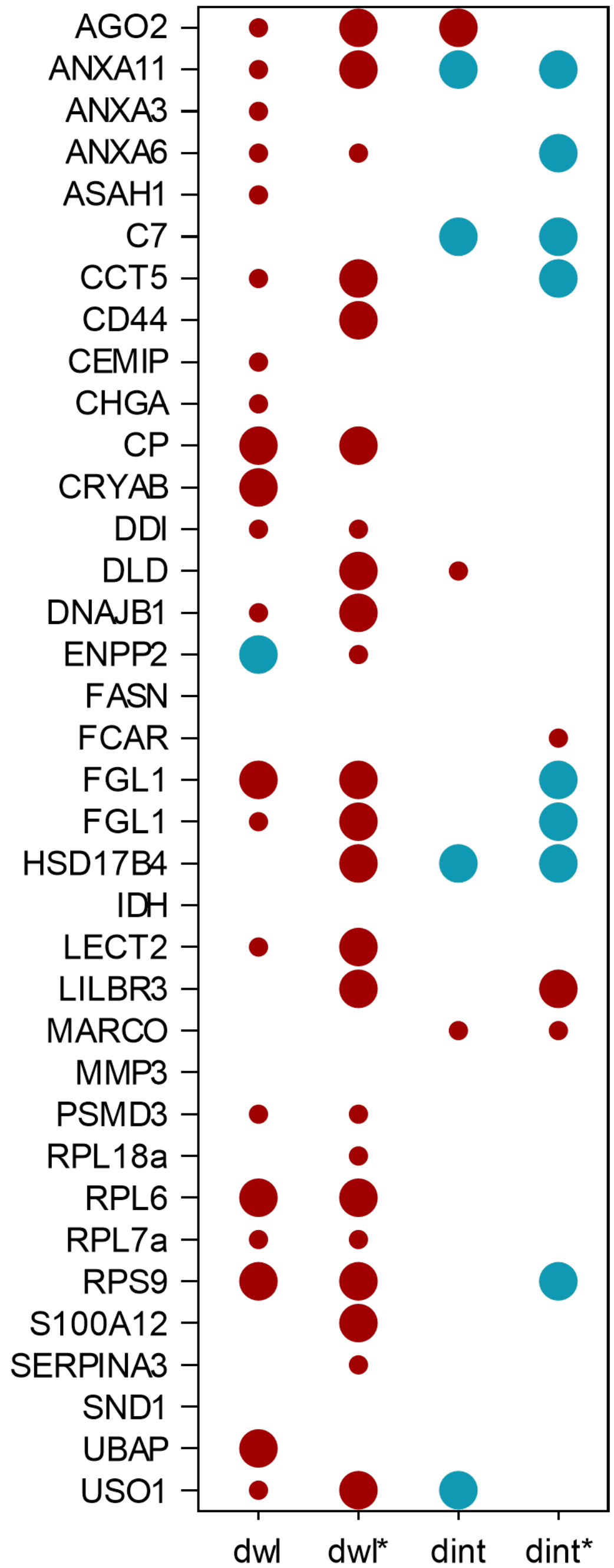

Figure S19. Fluorescence response of all DSPE-PEG wrapped 3,4,5-trifluoroaryl QWNTs proteins. Fluorescence titration data from Goerzen et al.[1] for the full protein panel, listed alphabetically. Each protein was titrated from 0.015 to 246 µg/mL. Circles mark significant responses; color gives direction (red, increase; blue, decrease) and size gives regime (small, near-baseline; large, linear). Only responses significant above a magnitude cutoff of 1 nm for the wavelength features and 20% for the intensity features are presented.

Table S1. Chemical parameters of QWDs in buffer protonation states. Six chemical descriptors of the aryl QWD categorized with the following physicochemical variable: electronic (Hammett constant), steric (polarizability, relative volume, hydrodynamic size), and thermodynamic (H-bond capacity, logP). Within the steric category, polarizability, relative volume, and hydrodynamic size (R_H) are nearly interchangeable, so we used R_H as a single representative steric descriptor in the main text analysis. H-bond capacity and logP are rank-correlated across the QWD series, and we group them as a single thermodynamic axis.

Relative volume: McGowan volume (cm^3/mol). R_H: Hydrodynamic radius derived using the standard Wilke-Chang Equation for diffusion in water, then converted diffusivity into hydrodynamic radius using the Stokes-Einstein equation. Hammett: Hammett substituent coefficient of terminating moieties of aryl QWDs. H-bond: Categorical rank of hydrogen bond donor capability of QWDs at pH 7.4 (high to low: H-bond acceptor to donor). Polarizability: Molar refractivity (cm^3/mol).

Terminating group of aryl QWD	Relative volume	R_H	Polarizability	Hammett	H-bond	logP	Charge
N,N-diethyl-4-amino	137.98	3.48	49.8	-0.83	1	3.7	0.126
3,5-dinitro	106.48	2.83	37.6	1.42	3	1.5	0
4-carboxyl	93.17	3.04	31	0	4	-2	-0.999
3,4,5-trifluoro	76.95	2.44	26.1	0.74	2	2.6	0

Table S2. Protein features analyzed in this study.

Category	Variable	Description
Global	# AAs	Number of total amino acids
	MW [kDa]	Molecular weight in kDa
	ASA: Sum	Absolute surface area
	RSA: Median	Relative solvent accessibility, median
	% Positive	Fraction of positively charged amino acids (R,H,K)
	% Negative	Fraction of negatively charged amino acids (D,E)
	% Polar	Fraction of polar amino acids (S,T,N,Q,C,Y,D,E,K,R,H)
	% Neutral Polar	Fraction of neutral, polar amino acids (S,T,N,Q,C,Y)
	% Nonpolar	Fraction of neutral, nonpolar amino acids (G,A,V,L,I,M,F,W,P)
	% Aromatic	Fraction of aromatic amino acids (F,Y,W,H)
	GRAVY	Grand Average of Hydropathy, a measure of overall hydrophobicity
	pI	Isoelectric point
Surface	% AAs Exposed	Fraction of exposed amino acids over all amino acids
	% Exp/Exp Positive	Fraction of positive exposed amino acids over all exposed amino acids
	% Exp/All Positive	Fraction of positive exposed amino acids over all amino acids
	% Exp/Exp Negative	Fraction of negative exposed amino acids over all exposed amino acids
	% Exp/All Negative	Fraction of negative exposed amino acids over all amino acids
	% Exp/Exp Polar	Fraction of polar exposed amino acids over all exposed amino acids
	% Exp/All Polar	Fraction of polar exposed amino acids over all amino acids
	% Exp/Exp Nonpolar	Fraction of nonpolar exposed amino acids over all exposed amino acids
	% Exp/All Nonpolar	Fraction of nonpolar exposed amino acids over all amino acids
	% Exp/Exp Aromatic	Fraction of aromatic exposed amino acids over all exposed amino acids
	% Exp/All Aromatic	Fraction of aromatic exposed amino acids over all amino acids
Structure	% Helix	Fraction of amino acids predicted to be part of an alpha helix
	% Strand	Fraction of amino acids predicted to be part of a beta strand
	% Other	Fraction of amino acids predicted to be part of a loop instead of an alpha helix or a beta sheet.
	SS: Helix	Secondary structure: Alpha Helix
	SS: Turn	Secondary structure: Turn
	SS: Sheet	Secondary structure: Beta Sheet
	Flex: S.D.	Flexibility, standard deviation
	Flex: Median	Flexibility, median

Table S3. Functionalization via in-situ production of diazonium.

Aniline	SG65i (mg)	Water (mL)	10x PBS (uL)	$NOBF_4$ (umol)	Aniline (umol)	H_2O_2 (50% wt. in H_2O)	Reaction Time
4-aminobenzoic acid<br>Sigma-Aldrich<br>Lot: 1003627281		0.5		4.2	4.2		
	0.8		100			200 uL	3 Days
3,4,5-trifluoroaniline<br>TCI Chemical<br>Lot: 78K8K-IK		0.7		4.48	4.48		

Table S4. Diazonium functionalization conditions for each QWNT type.

Diazonium	SG65i (2mg/mL)	10x PBS	Diazonium salt	H_2O_2 (50% wt. in H_2O)	Reaction Time
N,N-diethyl-4-aminobenzene diazonium tetrafluoroborate	400 uL		0.2 mg	200 uL	3 Days
		100 uL			
3,5 dinitrobenzene diazonium tetrafluoroborate	500 uL<br>+310 uL H_2O		0.36 mg	197 uL	1 Hour

Diazonium salts were synthesized following the protocol reported by Piao et al.[4] N,N-Diethyl-p-phenylenediamine (Sigma-Aldrich, Lot: STBK1127) and 3,5-dinitroaniline (Sigma-Aldrich, CAS-No: 618-87-1) were used as received.

Table S5. Protein specifications. All proteins were purchased from R&D Systems.

Protein	Gene symbol	UniProt accession	Length (aa)	Catalog no.	Lot no.	Source	Notes
Serine protease HTRA1	HTRA1	Q92743	480	1407-PI	IGO0224122	Recombinant human, E. coli derived	6-His tag, >95% purity
Insulin-like growth factor binding protein 3	IGFBP3	P17936	291	675-B3	BUY1325061	Recombinant human, NS0-derived	>95% purity
Lipoprotein lipase	LPL	P06858	475	2590-PGB	NLC1725081	Recombinant human, NS0-derived	6-His tag, >95% purity
Peptidoglycan recognition protein 1	PGLYRP1	O75594	196	292-G2	DS3025051	Recombinant human, E. coli derived	>97% purity
Insulin-like growth factor II	IGF2	P01344	180	7765-TG	DCNB0324121	Recombinant human, Sf 21-derived	6-His tag, >70% purity
Cathepsin D	CTSD	P07339	412	2916-SE	DAAJ0723121	Recombinant human, E. coli derived	6-His tag, >90% purity
Beta-1,4-mannosyl-glycoprotein 4-beta-N-acetylglucosaminyltransferase	MGAT3	Q09327	533	9085-SA	QCM0325021	Recombinant human, E. coli derived	>95% purity
Transglutaminase 1	TGM1	P22735	817	1014-AS	FSP0325101	Recombinant human, NS0-derived	10-His tag, >95% purity
Psoriasin	S100A7	P31151	101	9888-LL	DISO0525071	Recombinant human, CHO-derived	6-His tag
Cystatin A	CSTA	P01040	98	7359-GT	DCAY0625081	Recombinant human, CHO-derived	6-His tag, >90% purity

Table S6. Preparation of elution solutions for Thermo-Scientific TMT-labeled peptides.

Fraction Number	Percent of Acetonitrile
Second Wash	5.0
1	7
2	12
3	17
4	22
5	30
6	50

Supplementary References

1. Goerzen, D. et al. Machine perception liquid biopsy identifies brain tumours via systemic immune and tumour microenvironment signature. *Nat Nanotechnol* **21**, 277-287 (2026).

2. Pace, C.N. & Scholtz, J.M. A helix propensity scale based on experimental studies of peptides and proteins. *Biophys J* **75**, 422-427 (1998).

3. Chou, P.Y. & Fasman, G.D. Empirical predictions of protein conformation. *Annu Rev Biochem* **47**, 251-276 (1978).

4. Piao, Y. et al. Brightening of carbon nanotube photoluminescence through the incorporation of sp^3 defects. *Nat. Chem.* **5**, 840-845 (2013).
